# Long-term effects of adding an SGLT-2 inhibitor to insulin therapy in patients with type 1 diabetes. An observational study and systematic review of real-world evidence

**DOI:** 10.1007/s40618-025-02602-8

**Published:** 2025-05-10

**Authors:** Alberto Maran, Federico Boscari, Carlo Fagarazzi, Maria Cristina Crepaldi, Monica Vedovato, Benedetta Maria Bonora, Daniela Bruttomesso, Mario Luca Morieri, Gian Paolo Fadini

**Affiliations:** 1 Department of Medicine, University of Padova, Padova, Italy; 2 Division of Metabolic Disease and Diabetology, University Hospital of Padova, Via Giustiniani 2, Padua, 35128 Italy; 3 Veneto Institute of Molecular Medicine, Padova, Italy

**Keywords:** Gliflozin, Autoimmune, Adverse events, Persistence

## Abstract

**Purpose:**

The addition of SGLT2i to insulin therapy in type 1 diabetes (T1D) is an emerging treatment strategy. This study evaluates the real-world effects of SGLT2i on glycaemic control and other outcomes in individuals with T1D.

**Methods:**

In this single-center retrospective study, we included 78 adults with T1D who initiated SGLT2i and were observed for up to 24 months. Data included demographics, laboratory values, diabetic complications, and ongoing therapy. The primary outcome was the change in HbA1c over time. Persistence on therapy and adverse events were also recorded.

**Results:**

The mean age was 47.2 years, diabetes duration 24.6 years, baseline HbA1c 8.3%, and BMI 29.8 kg/m^2^. The median persistence on therapy was 14.8 months. HbA1c reduction was significantly associated with persistence (*p* = 0.01), with a maximum decrease of 0.61% at 6 months (*p* < 0.001). Time in range improved by 13.7% at 3 months (*p* < 0.001). Persistent users experienced a maximum weight loss of 2.5 kg at 9 months (*p* < 0.001). Insulin doses declined significantly (max 15% at 21 months). UACR declined significantly at 15 months (*p* = 0.025). Treatment discontinuation due to adverse events (mainly genitourinary tract infections) occurred in 25.6% of patients, and 1 episode of diabetic ketoacidosis was recorded. A review of the literature suggests that the observed effects are within the range of benefits reported previously from different countries.

**Conclusion:**

SGLT2i addition to insulin therapy in T1D patients resulted in sustained HbA1c reductions and weight loss. Therapy persistence significantly influenced outcomes, underscoring the importance of patient selection and monitoring for adverse effects.

## Introduction

Type 1 diabetes (T1D) management primarily relies on insulin therapy. However, despite the advancement in glucose monitoring and automated insulin infusion, achieving optimal glycaemic control remains challenging for many patients [1]. Unmet needs in T1D treatment include simplifying regimens, reaching HbA1c targets, reducing mental effort, and increasing time in range (TIR), without hypoglycaemia [2].

An ideal adjunct therapy to insulin would address these challenges by improving glycaemic control, reducing insulin requirements, aiding in weight management, and minimizing the risk of hypoglycaemia [3]. Sodium-glucose cotransporter-2 inhibitors (SGLT2i) have been explored as potential adjunctive treatments in T1D.

Clinical trials have evaluated the efficacy and safety of SGLT2i as adjunctive therapy to insulin in individuals with T1D [4]. These studies have demonstrated that SGLT2i can improve glycaemic control, reduce body weight, and decrease blood pressure.

The DEPICT (Dapagliflozin Evaluation in Patients with Inadequately Controlled Type 1 Diabetes) program, comprising two pivotal phase 3 trials, assessed dapagliflozin impact on glycaemic control in T1D patients. In these studies, participants receiving dapagliflozin 5 mg or 10 mg daily experienced significant reductions in HbA1c levels compared to placebo, along with weight loss and decreased insulin doses [5]. However, an increased incidence of diabetic ketoacidosis (DKA) was observed in the dapagliflozin groups. Based on these trials, in March 2019, dapagliflozin was approved by the EMA as an add-on treatment to insulin in adults with T1D and BMI ≥ 27 kg/m² who could not achieve adequate glycaemic control with insulin alone [6].

The EASE (Empagliflozin as Adjunctive to Insulin Therapy) trials investigated empagliflozin in T1D management. EASE-2 and EASE-3 demonstrated that empagliflozin, particularly at a 2.5 mg daily dose, led to modest HbA1c reductions and weight loss without a significant increase in DKA risk compared to placebo. Higher doses (10 mg and 25 mg) were associated with greater glycaemic benefits but also a higher incidence of DKA [7].

Real world studies are still limited, but overall they seem to confirm that SGLT2i therapy can offer metabolic benefits for T1D patients, though the associated risk of DKA necessitates careful patient selection, education, and monitoring to ensure safety [8].

Thus, despite possible benefits on glucose and weight management, concerns remain regarding the risk of DKA have led to regulatory actions. Notably, in October 2021, AstraZeneca voluntarily withdrew the indication of dapagliflozin for T1D in the European Union due to safety concerns [9].

Given these developments, our study aims to evaluate the real-world effects of SGLT2i therapy as an add-on to insulin in individuals with T1D. We focus on assessing glycaemic control, body weight, renal parameters, therapy persistence, and adverse events to provide insights into the practical benefits and risks of this adjunctive treatment approach.

## Methods

### Study design

This was a single-center retrospective study. The protocol complied with the Declaration of Helsinki and was part of the DiOR (Diabetes Outcome Research) protocol (422n/AO/23), approved by the ethical committee of the University Hospital of Padova. All patients provided written informed consent.

### Population

Patients were selected among those routinely attending the Diabetes Outpatient Clinic of the University Hospital of Padova. Inclusion criteria were: (i) a diagnosis of T1D since at least one year; age 18 years or older; (ii) add-on therapy with an SGLT2i on top of insulin therapy (multidose injections or insulin pump). Exclusion criteria were: age < 18 years, diagnosis other than T1D, missing data for key variables of interest.

### Exposure

The exposure was the therapy with SGLT2i added to insulin therapy. We considered the type of SGLT2i and the duration of therapy. The index data was set as the date patients received the first prescription of SGLT2i. We then evaluated the persistence on therapy during subsequent visits as reported in the electronic chart system. There was no control on adherence and persistence based on pharmacy refill rates or medicine possession ratio.

### Data collection

For all patients, we recorded the following data by automated extraction from the electronic health records: demographics (age, sex, diabetes duration), anthropometrics (weight, and height for the calculation of BMI), other cardiovascular risk factors (blood pressure, diagnosis of hypertension, smoking habit), Laboratory data (HbA1c, fasting glucose, serum lipids, serum creatinine for the calculation of eGFR [estimated glomerular filtration ratio] with the CKD-EPI equation, urinary albumin/creatinine ratio [UACR]), diabetic complications and ongoing therapy. Complications were defined as follows: micro- and macroalbuminuria were defined using the traditional 30 and 300 mg/g cut-off; CKD stage III or greater was defined as an eGFR < 60 ml/min/1.73 m^2^; retinopathy (including maculopathy) was defined based on digital ophthalmologic examination; neuropathy (including somatic or autonomic) was defined based on clinical history, questionnaires, physical examination and instrumental diagnosis (nerve conduction velocity and/or cardiac autonomic tests). Microangiopathy was defined as any among raised albuminuria, reduced eGFR, retinopathy or neuropathy. Coronary artery disease was defined as a history of myocardial infarction, or angina, or coronary revascularization; cerebrovascular disease was defined as a history of stroke or transient ischemic attack or cerebral artery revascularization; peripheral arterial disease was defined as a history of claudication or rest pain or peripheral revascularization; carotid atherosclerosis was defined in the presence of ultrasound evidence of carotid plaques. Cardiovascular disease was defined as coronary, cerebral or peripheral artery disease, while macroangiopathy also included carotid atherosclerosis (even if asymptomatic).

### Outcomes

Outcomes were evaluated at each subsequent visit after the index date. Visit dates were forced into 3-months intervals up to the last available evaluation. The primary outcome was the change in HbA1c over time after add-on of SGLT2i to insulin therapy. HbA1c measure was IFCC-aligned but not centralized and collected as recorded in the electronic records. Secondary outcomes included the change in body weight, blood pressure, lipids, eGFR and UACR, and the change in CGM (continuous glucose monitoring) metrics, when available. We also recorded whether or not the patient was still taking SGLT2i and eventual side effects and reasons for discontinuation (safety outcomes).

### Review of the literature

The following search string was run in Pubmed: (“type 1 diabetes”) and (“SGLT2” or “SGLT-2” or “glycosuric” or “gliflozin” or “dapagliflozin” or “empagliflozin” or “canagliflozin” or “ipragliflozin” or “sotagliflozin” or “ertugliflozin”) and (“observational” or “real-life” or “real-world” or “retrospective”). We then retained only original articles reporting efficacy or safety outcomes in patients with T1D who received add-on therapy with SGLT2i. We extracted relevant information on the population under investigation and on the following outcomes: change in HbA1c, body weight, CGM metrics, and DKA.

### Statistical analysis

Continuous data are presented as mean (standard deviation), while categorical variables are presented as number (percentage). Markedly skewed variables (e.g. UACR) are reported as median (interquartile range, IQR), as were observation times. The change over time in continuous variables (including HbA1c, the primary endpoint) was analysed using the mixed model for repeated measures (MMRM). The model specified the dependent variable (the outcome variable, e.g. HbA1c), time in months, the baseline value of the dependent variable (e.g. baseline HbA1c) as fixed factors and eventual subgroups (e.g. by sex, obesity status), group-by-time interactions, or covariates. Persistence on drug was entered in the model as a time-varying fixed factor. The first-order autoregressive variance structure was chosen. The model output included the estimated mean at each time point, the overall effect, the eventual mean estimated differences between groups and the significance of the time-by-group interaction. Adjustment for multiple testing was performed only within each model’s timepoints with LSM, but not across the various outcome analysis. SPSS version 23 was used and statistical significance was accepted at the conventional p value < 0.05.

## Results

### Baseline characteristics of study patients

We included 78 patients with T1D (61.5% women), the majority of whom were on multidose insulin, and only 9 (11.5%) were on insulin pump. Mean age was 47.2 years and the mean duration of diabetes was 24.6 years. Mean BMI was 29.8 kg/m^2^ and only 12 patients (15.4%) were normal weight. Baseline HbA1c was 8.3% and fasting glucose 182.4 mg/dl. The mean (SD) HbA1c in the 12 months before index date was 8.3% (1.0). Two out of three patients (66.7%) had hypertension (mostly on a RAS blocker), while smokers were only 12.8%. eGFR was overall preserved (mean 96.9 ml/min/1.73 m^2^) and only 9% of patients had a urinary-albumin creatinine ratio ≥ 30 mg/g. 60.3% of patients had microangiopathy (the most common being retinopathy), while 29.5% had macroangiopathy (the most common being carotid atherosclerosis). The summary of characteristics is illustrated in Table 1.

**Table 1:** Baseline characteristics of study participants

Variable	Values
**Demographics**	*n* = 78
Women, n (%)	48 (61.5)
Age, years	47.2 (15.4)
Diabetes duration, years	24.6 (13.5)
**Anthropometrics**	
Weight, kg	84.5 (15.0)
BMI, kg/m^2^	29.8 (4.4)
**Risk factors**	
Systolic blood pressure, mm Hg	135.2 (15.7)
Diastolic blood pressure, mm Hg	77.5 (12.0)
Hypertension, n (%)	52 (66.7)
Smoke, n (%)	10 (12.8)
**Laboratory data**	
HbA1c, %	8.3 (1.1)
Fasting glucose, mg/dl	182.4 (72.2)
Total cholesterol, mg/dl	167.5 (31.3)
HDL cholesterol, mg/dl	58.0 (15.0)
LDL cholesterol, mg/dl	90.9 (26.5)
Triglycerides, mg/dl	93.2 (51.5)
eGFR, ml/min/1.73 m^2^	96.9 (21.9)
UACR, mg/g Median (IQR)	44.5 (179.7) 6.05 (2.9–10.4)
**Complications**	
Microangiopathy, n (%)	47 (60.3)
Microalbuminuria, n (%)	4 (5.1)
Macroalbuminuria, n (%)	3 (3.8)
CKD stage 3+, n (%)	6 (7.7)
Retinopathy, n (%)	36 (46.2)
Neuropathy, n (%)	30 (38.5)
Macroangiopathy, n (%)	23 (29.5)
Cardiovascular disease, n (%)	9 (11.5)
Coronary artery disease, n (%)	9 (11.5)
Cerebrovascular disease	0 (0.0)
Carotid atherosclerosis, n (%)	16 (20.5)
Peripheral arterial disease, n (%)	11 (14.1)
**Therapies**	
Metformin, n (%)	16 (20.5)
Insulin pump, n (%)	9 (11.5)
RAS blockers, n (%)	38 (48.7)
Hypolipidemic therapy, n (%)	46 (59.0)

Data are presented as mean (SD) for continuous variables and number (%) for categorical variables

SGLT2i were distributed as follows: dapagliflozin 5 mg (51.9%), dapagliflozin 10 mg (24.7%), empagliflozin (16.9%, equally distributed on 10 mg and 25 mg), and canagliflozin (6.5%, mostly 100 mg or 150 mg). The total baseline insulin dose was 0.63 U/kg.

### Persistence and tolerability

The median (IQR) observation time was 20 (2.7–40.7) months and the median (IQR) persistence on drug was 14.8 (3.3–38.3) months. *N* = 23 patients (29.5%) experienced adverse events, including 18 (23.1%) genitourinary tract infections; *n* = 1 DKA (rate 0.7/100 patients-year; 95% C.I. 0.1-4.0), *n* = 1 severe hypoglycaemia; *n* = 4 adverse events not otherwise specified. The DKA episode occurred in a female patient with T1D and obesity, who was taking canagliflozin since > 12 months with concomitant reduction in insulin doses by almost 50%. She was on CGM but was not checking capillary ketones regularly; the episode was precipitated by gastroenteritis and fully recovered. Canagliflozin was stopped at DKA presentation and not re-introduced after resolution. In the subsequent 7 years, no other DKA events were reported in the same patient.

Adverse events led to discontinuation in *n* = 20 patients (25.6%). The rate of discontinuation was not significantly different between men and women (HR 1.80; 95% C.I. 0.49–6.81; *p* = 0.384) and was not associated with obesity or complications. After discontinuation due to an adverse event, therapy with SGLT2i was not re-introduced.

### Effect on glycaemic control

**Fig. 1:**
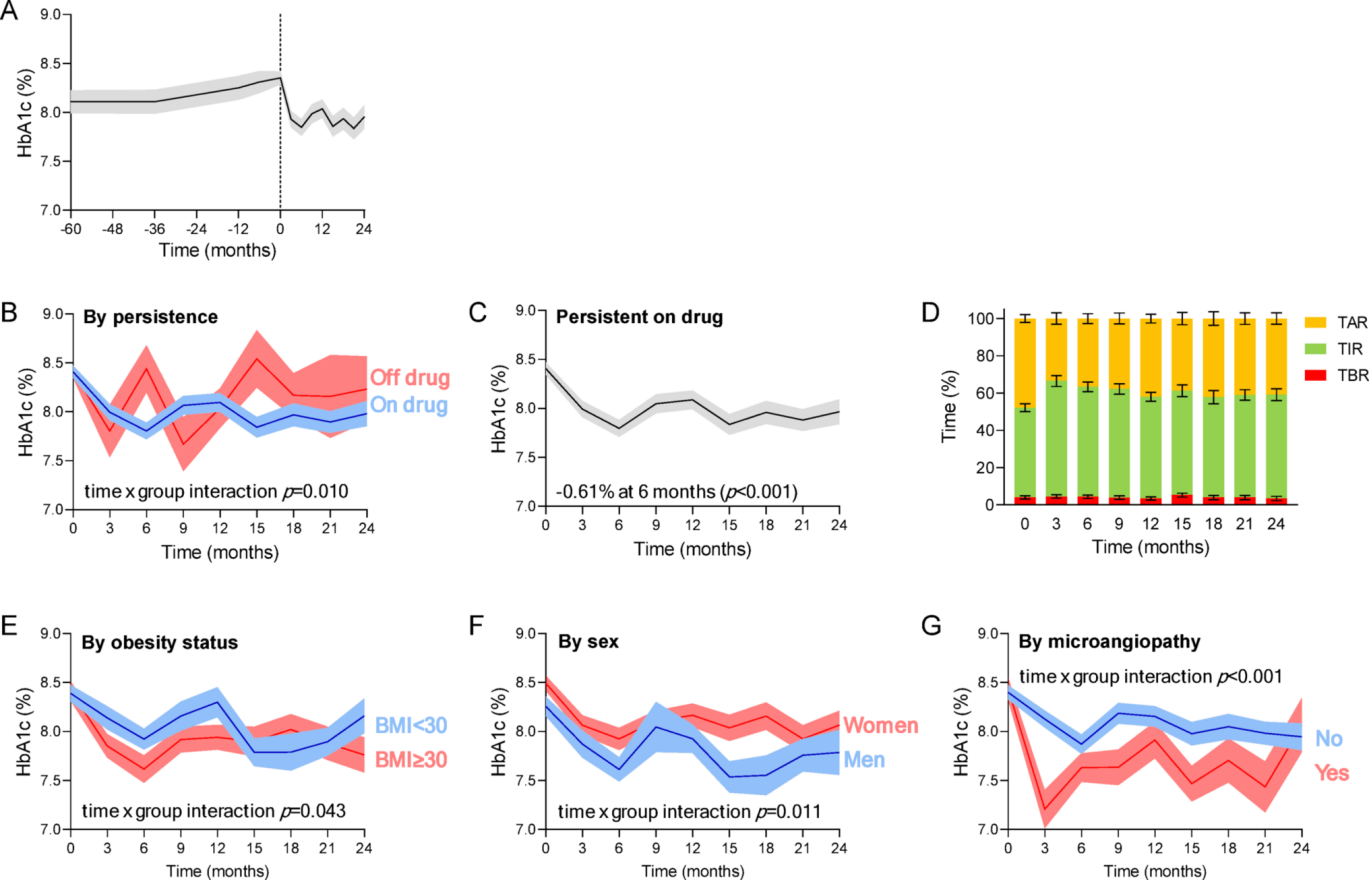
Glycaemic effects. **A**) Change in HbA1c in the 5 years before and in the 2 years after index date (dashed line at time zero), i.e. the date of initiation of SGLT2i. **B**) Change in HbA1c according to persistence on SGLT2i. **C**) Change in HbA1c analysed with SGLT2i on board (persistent on drug). **D**) Glucose metrics from the continuous glucose monitoring system (TIR, time in range. TBR, time below range. TAR, time above range). Bars indicate standard errors. **E**) Change in HbA1c in patients who persisted on drug and divided by obesity status (BMI below or greater/equal than 30 kg/m^2^). **F**) Change in HbA1c by sex. **G**) Change in HbA1c by presence or absence of microangiopathy. The curves from mixed model for repeated measures (MMRM) illustrate the mean effect while the shaded area indicate the standard error. The time by group interaction p-value in the MMRM model is also reported

During the observation, no patient changed the insulin treatment strategy (MDI, pump or AID). The change in HbA1c over time was analysed with the mixed model for repeated measures, considering all available visits and adjusting for baseline HbA1c and persistence on drug. While glycaemic control remained insufficient and showed a trend worsening in the 5 years before index date, HbA1c rapidly declined after initiation of SGLT2i and remained lower than the baseline value for up to 24 months (Fig. 1A). HbA1c reduction was significantly different according to drug persistence (time-by-persistence interaction *p* = 0.01; Fig. 1B). Considering the effect on-drug, the maximum HbA1c reduction was observed at 6 months (-0.61%; *p* < 0.001; Fig. 1C). Time in range (TIR, available for 58% of patients) was significantly improved already at 3 months (+ 13.7%; 95% C.I. from 7.7 to 19.7%; *p* < 0.001) and remained significantly higher than at baseline up to month 24. Time below range (TBR) was not significantly modified, while, as expected, time above range (TAR) decreased at 3 months (-15.0%) and remained lower for up to 24 months (Fig. 1D).

Nominally, the change in HbA1c was significantly greater, in participants with obesity (BMI ≥ 30 kg/m^2^) than in those without, in men than in women, and in the presence of microangiopathy (Fig. 1E-G). However, only sex remained independently associated with the change in HbA1c at the multivariable analysis (Table 2): women had an average 0.26% higher HbA1c during the entire observation (*p* = 0.025) and displayed a significantly lower glycaemic effect after adding SGLT2i (time-by-sex interaction *p* = 0.011).

**Table 2:** Multivariable analysis of factors associated with glycaemic and weight response

	Change in HbA1c			Change in body weight		
Covariate	Estimate	SE	*P*	Estimate	SE	*P*
Age	− 0.001	0.01	0.920	− 0.02	0.06	0.859
Female	0.26	0.12	0.025	0.06	0.07	0.433
Diabetes duration	0.0001	0.01	0.978	− 0.11	0.08	0.958
Macroangopathy	− 0.11	0.15	0.442	− 0.06	2.29	0.869
Microangiopathy	− 0.06	0.18	0.731	0.50	1.29	0.703
BMI	0.01	0.03	0.570	0.51	2.34	0.830
eGFR	0.002	0.01	0.600	0.05	0.07	0.473

Covariates were entered in the MMRM model for HbA1c and body weight change over time. Time and baseline HbA1c / body weight were entered as fixed factors. For each analysis the estimate changes in HbA1c and weight associated with the covariate are presented along with their standard error (SE) and P value

### Effect on body weight

**Fig. 2:**
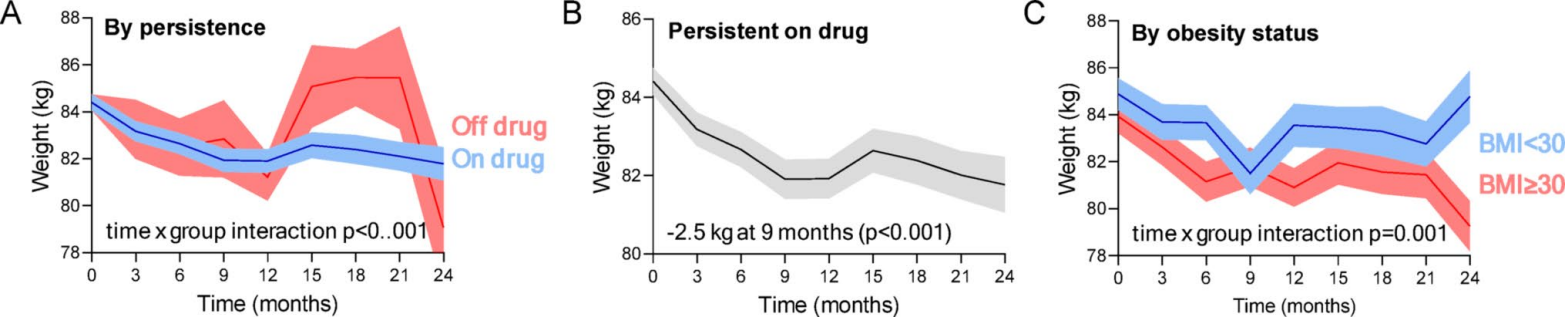
Effects on body weight. **A**) Change in body weight according to persistence on SGLT2i. **B**) Change in body weight analysed with SGLT2i on board (persistent on drug). **C**) Change in body weight in patients who persisted on drug and divided by obesity status (BMI below or greater/equal than 30 kg/m^2^). The curves from mixed model for repeated measures (MMRM) illustrate the mean effect while the shaded area indicate the standard error. The time by group interaction p-value in the MMRM model is also reported

The change in body weight was significantly different according to persistence on drug, with non-persistent patients showing an increase in body weight after 12 months (Fig. 2A). Among participants who persisted on drug, the maximum weight loss was observed at 9 months and was − 2.5 kg (*p* < 0.001; Fig. 2B). Weight loss appeared to be more pronounced in patients with obesity at baseline (time-by-obesity interaction *p* = 0.001; Fig. 2C). However, no clinical variable remained independently associated with weight loss in the multivariable analysis (Table 2).

### Other endpoints

**Fig. 3:**
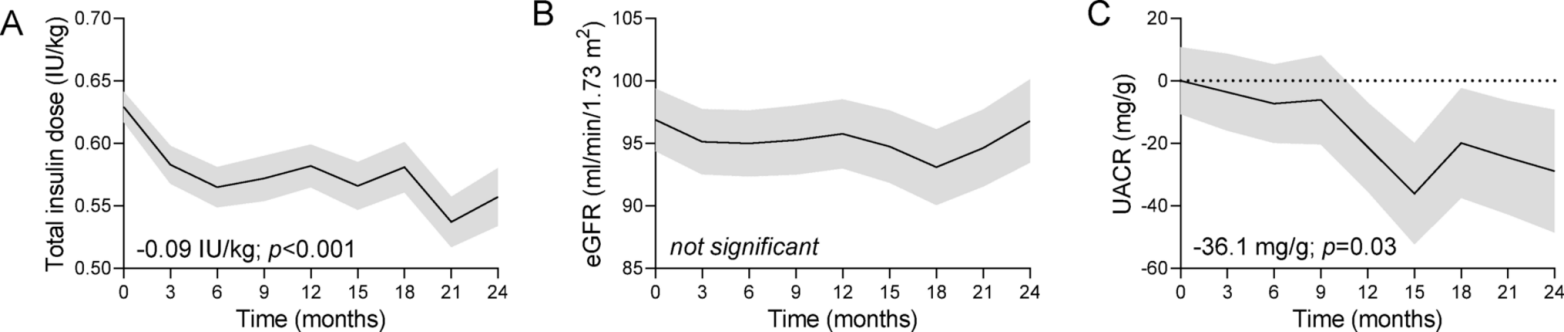
Other outcomes. **A**) Insulin requirements (total international units / kg body weight) over time. **B**) Change over time in estimated glomerular filtration rate (eGFR). **C**) Change over time in urinary albumin/creatinine ratio (UACR). The curves from mixed model for repeated measures (MMRM) illustrate the mean effect while the shaded area indicate the standard error

Total insulin requirement decreased significantly from baseline up to 24 months: the decline was already significant at 3 months and was maximal at 21 months (-0.09 U/kg; *p* < 0.001), equal to a 15% reduction from baseline (Fig. 3A).

The change in systolic and diastolic blood pressure was not statistically significant at any time point, but the concomitant change in blood pressure-lowering medications was not recorded. There were no statistically significant of clinical meaningful changes in lipid parameters (not shown). The change in eGFR was also not significant over time (Fig. 3B). Though a minority of patients had micro- or macroalbuminuria at baseline, UACR declined significantly by -36.1 (16.1) mg/g at 15 months (*p* = 0.025 after adjusting for multiple time point comparisons; Fig. 3C).

### Review of prior observational studies

Based on a literature search, we identified 15 retrospective studies reporting the change in glycaemic and extra-glycaemic endpoints after adding SGLT2i to insulin therapy in people with T1D [10–24]. Data are summarized in Table 3. The change in HbA1c (11 studies, 1018 patients) ranged from − 0.3% to -1.0% most likely in relation to baseline HbA1c (between 7.4% and 9.4%). The change in body weight (11 studies, 1018 patients) ranged from − 0.4 kg to -4.5 kg. Four studies (*n* = 315 patients) reported the change in TIR, which was quite consistent (from + 9.3% to + 11.6%), whereas the change in TBR was minimal and poorly consistent across studies. One study reported a significant reduction in median (IQR) UACR of -99 (36; 218) mg/g. The incidence of DKA was reported in 14 studies (*n* = 5354 patients), ranging from 0 to 10.8% or from 6.6 to 20.2 cases/100 patient-year.

**Table 3:** Literature review. Summary of the articles retrieved using the strategy described in the text. Continuous data are presented as mean SD or as median (IQR), while categorical variables are presented as number and percentages

References	Country	*n*	Age (years)	Female (*n* [%])	Diabetes duration (years)	BMI (kg/m2)	HbA1c (%)	MDI (*n* [%])	SGLT2i	Follow up	Weight Delta (kg)	HbA1c Delta (%)	TDI dose Delta (UI)	TIR Delta (%)	DKA
[12]	Spain	250	46.1 ± 12.7	133 (57.8)	26.4 ± 12.2	30.2 ± 4.8	7.9 ± 0.9	206 (82.4)	Dapa	12 mo	− 3.3	− 0.6	− 8.7	9.3	2.4% (CI 0.3;4.5)
[23]	Denmark	134	51.4 ± 13.6	72 (53.7)					Dapa						0%
[15]	Saudi Arabia	37	25.8 ± 8.0	22 (59.5)	10 ± 6.5	28.1 ± 6.7	9.4 ± 1.4	27 (100)	Empa	15.8 ± 6.0 mo	− 1.7 ± 5.88	− 0.82 ± 1.35	− 2.9 ± 5.9		10.80%
[20]	England	18		40.6 ± 12.7	24.0 ± 9.4	35 ± 5	8.9 ± 1.0	7 (39)	Dapa 5 mg	6 mo	− 2.8 (CI − 1.3; 6.8)	− 1.0 (CI 0.6; 1.4)	− 10.2		0%
[21]	Japan	12	50.1 ± 13.2	7 (58)	17.3 ± 10.5	22.9 ± 2.1	8.8 ± 1.3	12 (100)	Ipra	6 mo	− 1.4 ± 1.4	− 0.6			0%
[11]	Spain	27	45 ± 12	14 (63)	23 ± 12	32 ± 4	8.0 ± 0.7	22 (82)	Empa	52 wks	− 8	− 0.8		12	1 episode
[16]	USA	2304							Cana, dapa, or empa						7.12 (5.64–8.87)
[19]	Spain / Belgium	199	48.13 ± 10.11	113 (56.8)	25.5 ± 11.2	28.9 ± 3.7	8.2 ± 0.88	134 (67.3)	Cana, dapa, or empa	12 mo	− 2.9	− 0.5			7 (3.5)
[13]	USA	32	46.8 ± 14.4	14 (43.7)	22.0 ± 15.3		7.9 (7.5–8.4)	9 (28.1)	Cana, dapa, or empa	12 mo	− 1.7	− 0.6	− 1		12.8%; 6.6 events/100 PY
[17]	Japan	76	54.5 (45.0,63.3)	47 (61.8)	11.0 (4.8,23.3)	23.0 (20.9,24.8)	8.0 (7.4,9.1)	67 (88.2)	Dapa	24 mo		− 0.30 (− 0.80; 0.30)	− 2.5 (− 7.0; 0.0)		2 cases (0.3%)
[18]	Japan	1898	55.8 ± 15.0	998 (52.6)		24.5 ± 4.9		1,767 (93.1)							7.3%; 20.2 /100 PY
[24]	Japan	15	51.8 ± 15.7	8 (53.3)	15.2 ± 10.9	24.3 ± 3.4	8.9 ± 1.1	14 (93.4)	Ipra 50 mg or dapa 5 mg	> 3 wks	− 1.2		− 2	11.6	
[14]	USA	26	47 (23–67)	17 (65)	28	29.81 22–47	8.27 (6.4–10.7)	12 (46.1)	Cana, empa, or dapa	7.5 mo	− 0.4	-0.37			0
[22]	Portugal	23	45.9 ± 11.6	14 (60.9)	27.7 ± 11.7	26.0 (3.0)	7.4 ± 0.8	0 (0)	Dapa 5 mg, empa 5 mg, ertu 15 mg	12 mo	− 4.5	− 0.5	− 0.9	11.1	8.70%
[10]	Germany	318	53.0 (42.2; 61.6)	134 (42.1)	16.2 (8.6; 26.9)	30.1 (26.1; 34.5)	7.9 (7.2; 8.8)	236 (74.2)	dapa and empa	2.5 years	0.4 (− 0.5; 1.3)	− 0.3 (− 0.5; − 0.2)	− 2.9 (− 7.8; 2.1)		0

## Discussion

In our study involving 78 individuals with T1D, the addition of an SGLT2i to insulin therapy resulted in significant long-term improvements in glycaemic control and body weight. The maximum reduction in HbA1c was observed at 6 months (-0.61%), with sustained benefits up to 24 months. TIR increased by 13.7% at 3 months and remained elevated throughout the study period. Participants who maintained SGLT2i therapy experienced a maximum weight loss of 2.5 kg at 9 months. Adverse events were reported in 29.5% of patients, with genitourinary infections being the most common. There was one case of DKA, and 25.6% of participants discontinued the therapy due to adverse events.

The findings suggest that SGLT2i can be an effective adjunct to insulin in T1D management, offering improvements in glucose and weight management. However, the occurrence of adverse events, particularly genitourinary infections and the risk of DKA, underscores the necessity for careful patient selection and monitoring. The incidence of GUTI in our study is consistent to that reported in a European two-cohort study of people with T1D using SGLT2i [19]. The discontinuation rate due to adverse events highlights the importance of individualized treatment plans and patient education [8]. Indeed, while SGLT2i therapy provides sizeable metabolic benefits, these must be balanced against the potential risks. The risk of adverse events, especially DKA, necessitates vigilant monitoring and patient education to mitigate these risks [25]. The decision to incorporate SGLT2i into T1D treatment should involve a thorough discussion between healthcare providers and patients, weighing the benefits against the potential harms. Of note, the concerns on SGLT2i-assocated DKA does not only involve T1D, but also type 2 diabetes, especially elderly ones with ling-standing disease, who may develop beta-cell insufficiency [26,27].

Our results align with previous clinical trials that have demonstrated the efficacy of SGLT2i as an adjunctive therapy in T1D. Studies have reported moderate reductions in HbA1c, decreased body weight, and increased TIR without a significant rise in hypoglycaemia. However, these benefits are accompanied by an increased risk of DKA. The DEPICT and EASE trials reported similar findings, with glycaemic improvements offset by a higher incidence of DKA in treatment groups [5,7].

Other real-world studies corroborate our findings, indicating that SGLT2i use in T1D leads to reductions in HbA1c and body weight. Specifically, our results align with three large retrospective studies reporting the clinical effectiveness of SGLT2i in T1D [10,12,19], showcasing HbA1c improvements of around about 0.5% and weigh loss of about 3 kg. Interestingly, a report on the effects of dapagliflozin discontinuation after label withdrawal for T1D has documented a substantial deterioration of glucose, weight control and insulin doses after 3–6 months [28]. Yet, the risk of DKA remains a significant concern, with the largest real-world study reporting a rate of 7 cases per 100 patients-year [16]. In our study, the DKA rate was lower (although with large uncertainty, as only one episode was reported), probably because patients were provided with capillary ketone meters and/or educated on the sick-day rules and repeatedly instructed to monitor closely the occurrence of DKA symptoms.

Our findings suggest that certain subgroups of patients may derive greater benefits from SGLT2i therapy. Notably, individuals with BMI-based obesity appeared to respond better in terms of glycaemic improvement or weight loss. Additionally, men demonstrated a greater HbA1c reduction compared to women, although the mechanism of this finding is unclear and not supported by prior observations. Identifying these “best responders” can help personalize treatment strategies, maximizing benefits while minimizing risks.

A key point that remains largely unaddressed is whether SGLT2i exert cardio-renal protection in people with T1D. Our result of a significant improvement in UACR is aligned with a post-hoc analysis of the DEPICT trials for dapagliflozin [29], but studies with empagliflozin and sotagliflozin have not confirmed such an effect on UACR [30,31]. The absence of a significant decline in eGFR over 24 months following SGLT2i initiation may even suggest a protective effect against kidney function loss, given that eGFR typically declines with age (by approximately 0.75 to 1 mL/min/1.73 m² per year after age 40), and more steeply in the presence of diabetes [32,33]. However, in the absence of a control group, no definitive conclusion can be drawn on this endpoint.

While we had no opportunity to examine cardiac biomarkers or cardiovascular outcomes, it is important to remark that T1D increases the risk of heart failure by almost 3-times thereby representing an underappreciated complication in this relatively young population [34]. Emerging real-world data seem to confirm that people with T1D who received SGLT2i had a preservation of kidney function and a reduced risk of heart failure [35], but further studies are eagerly needed in support of this concept. Future research should also focus on refining selection criteria and implementing risk mitigation strategies, such as patient education and close metabolic monitoring, to enhance the safety and efficacy of SGLT2i in T1D management. The advent of continuous ketone body monitoring [36] may make SGLT2i safer in T1D and, along with the emergent evidence of cardio-renal protection, may legitimate a broader use of this class of medications in T1D. Another hot topic for future investigation is the use of SGLT2i for kidney protection in islet and/or kidney transplant recipients [37,38].

This study strengths include a relatively long follow-up period of up to 24 months, allowing for the assessment of both short-term and sustained effects of SGLT2i therapy. Indeed, most studies in our literature search had a shorter observation time. The real-world setting enhances the generalizability of the findings, reflecting routine clinical practice. Additionally, the study provides insights into patient persistence with therapy and the practical challenges encountered in managing T1D with adjunctive SGLT2i treatment.

The retrospective design may introduce selection bias and limits the ability to establish causality. The smaller-than-expected proportion of T1D patients on pump therapy [39] represents a selection bias, which may be explained by two factors: (i) insulin pump therapy often enables better glycaemic control, reducing the perceived need for add-on SGLT2 inhibitors; and (ii) concerns about an increased risk of DKA in pump users may discourage clinicians from prescribing SGLT2 inhibitors in this population. The relatively small sample size and the single-center setting may affect the generalizability of the results. Furthermore, the reliance on medical records from routine care may result in incomplete data, particularly concerning adverse events and patient adherence. The lack of a control group also limits the ability to attribute observed effects solely to SGLT2i therapy. Comparing HbA1c in the period before and after initiation of SGLT2i allowed us to rule out that the observed effect reflected spontaneous oscillations of HbA1c due to seasonality or other factors. Although we could not rule out the placebo effect, the sustained improvement of HbA1c up to 24 months seems to go beyond the typically transient improvements in the course of chronic diseases that occur after any change in therapy, However, we acknowledge that studies with a longer observation are needed to carefully scrutinize the risk/benefit ratio of this therapy.

In summary, our study supports the adjunctive use of SGLT2i in T1D management, demonstrating improvements in glycaemic and weight management. However, the associated risks need careful patient selection, comprehensive education, and close monitoring. These findings are consistent with existing literature from both clinical trials and real-world studies. Further research is warranted to identify strategies to mitigate risks and to determine which patient populations may benefit most from this therapy.
